# Safety and Feasibility of Tele-Supervised Home-Based Transcranial Direct Current Stimulation for Major Depressive Disorder

**DOI:** 10.3389/fnagi.2021.765370

**Published:** 2022-02-02

**Authors:** Davide Cappon, Tim den Boer, Caleb Jordan, Wanting Yu, Alexander Lo, Nicole LaGanke, Maria Chiara Biagi, Pawel Skorupinski, Giulio Ruffini, Oscar Morales, Eran Metzger, Bradley Manor, Alvaro Pascual-Leone

**Affiliations:** ^1^Hinda and Arthur Marcus Institute for Aging Research at Hebrew SeniorLife, Boston, MA, United States; ^2^Deanna and Sidney Wolk Center for Memory Health at Hebrew SeniorLife, Boston, MA, United States; ^3^Department of Neurology, Harvard Medical School, Boston, MA, United States; ^4^Neuroelectrics, Barcelona, Spain; ^5^Department of Psychiatry Harvard Medical School, Boston, MA, United States; ^6^Department of Medicine, Harvard Medical School, Boston, MA, United States; ^7^Guttmann Brain Health Institute, Barcelona, Spain

**Keywords:** transcranial direct current stimulation (tDCS), depression, home-based intervention, non-invasive brain stimulation, COVID-19

## Abstract

Major depressive disorder (MDD) is a worldwide cause of disability in older age, especially during the covid pandemic. Transcranial direct current stimulation (tDCS) is a non-invasive neuromodulation technique that has shown encouraging efficacy for treatment of depression. Here, we investigate the feasibility of an innovative protocol where tDCS is administered within the homes of older adults with MDD (patient participants) with the help of a study companion (i.e. caregiver). We further analyze the feasibility of a remotely-hosted training program that provides the knowledge and skills to administer tDCS at home, without requiring them to visit the lab. We also employed a newly developed multi-channel tDCS system with real-time monitoring designed to guarantee the safety and efficacy of home-based tDCS. Patient participants underwent a total of 37 home-based tDCS sessions distributed over 12 weeks. The protocol consisted of three phases each lasting four weeks: an acute phase, containing 28 home-based tDCS sessions, a taper phase containing nine home-based tDCS sessions, and a follow up phase, with no stimulation sessions. We found that the home-based, remotely-supervised, study companion administered, multi-channel tDCS protocol for older adults with MDD was feasible and safe. Further, the study introduces a novel training program for remote instruction of study companions in the administration of tDCS. Future research is required to determine the translatability of these findings to a larger sample.

**Clinical Trial Registration:** https://clinicaltrials.gov/ct2/show/NCT04799405?term=NCT04799405&draw=2&rank=1, identifier NCT04799405.

## Introduction

Major depressive disorder (MDD) is highly prevalent and a leading cause of disability worldwide (Kupfer et al., 2012). Pharmacological treatments have limited efficacy, side effects are common, and one-third of patients are medication-resistant, failing to achieve remission after using three or more antidepressants (Rush et al., 2006; Nemeroff, 2007). Older age is a significant predictor of an unfavorable course of depression (Mitchell and Subramaniam, 2005), reduced likelihood of treatment response (Licht-Strunk et al., 2007; Tedeschini et al., 2011), and increased risk of relapse (Beekman et al., 2002).

For patients with medication-resistant MDD, a few neuromodulation methods are available, including electro-convulsive therapy (ECT) and repetitive transcranial magnetic stimulation (rTMS). These are effective interventions; however, they require access to suitable clinic facilities, which was recently challenging due to the COVID-19 pandemic restrictions. At the same time, the COVID-19 pandemic has increased the risk of depression due to social isolation, loneliness, high stress and fear of infection. There is thus an urgent need for a safe, effective, home-based intervention for acute, medication-resistant episodes of uni- or bi-polar MDD.

Transcranial direct current stimulation (tDCS) is a neuromodulation technique that provides a possible solution. In tDCS, surface electrodes (anode and cathode) inject low amplitude direct current through the scalp and brain. tDCS is safe and well-tolerated when appropriate safety guidelines are followed (Antal et al., 2017). The establishment of the antidepressant efficacy of tDCS has proven challenging. Although there are numerous randomized controlled trials (RCTs) that demonstrated tDCS to be superior to sham stimulation (placebo) in treatment of depression (Loo et al., 2012; Brunoni et al., 2013, 2017; Sampaio-Junior et al., 2018), a number of RCTs failed to find such an effect (Loo et al., 2010, 2018; Palm et al., 2012; Bennabi et al., 2015). To clarify this discrepancy in the literature, two recent meta-analyses, including 23 and 9 RCTs respectively, concluded that tDCS was indeed superior to sham regarding endpoint depression scores, response, and remission rates, but that the effect size was moderate (Moffa et al., 2020; Razza et al., 2020). Crucially, Moffa et al. (2020) presented evidence that longer treatment courses could lead to an enhancement of the efficacy of tDCS.

Several equipment manufacturers have recently developed portable tDCS systems that are particularly well-suited for remotely supervised stimulation at home, which would facilitate longer periods of treatment, especially for people living within remote settings and when in-person clinical visits are not possible. Recently, there has been a significant increase in research interest for the application of home-based tDCS to a breadth of clinical conditions (for review, please refer to Palm et al., 2018; Sandran et al., 2019; Gough et al., 2020). In Table 1 we summarized some of the features of the published randomized controlled trials employing the home-based tDCS approach. In the context of depression, Alonzo et al. (2019) completed an open-label trial of home-based tDCS in 34 participants that self-administered tDCS sessions over four weeks followed by a taper phase. While that study reported clinical improvement at the group level, the authors highlighted several limitations and challenges.

**TABLE 1:** Randomized controlled trials adopting home-based tDCS.

Citation	Article Type	Stim Type	Clinical Population	Sample Size	Number of Sessions
Ahn et al., 2019	Double blind RCT	tDCS	Knee Osteoarthritis	30	10
Ahn et al., 2020	Double blind RCT	tDCS	Knee Osteoarthritis	30	10
Brietzke et al., 2020	Double blind RCT	tDCS	Fibromyalgia	20 (10 active/10 sham)	60
Brooks et al., 2021	Double blind RCT	tDCS	Geriatric Depression or Anxiety	26 (12 active/14 sham)	50 (5/week)
Carvalho et al., 2018	Double blind RCT	tDCS	Healthy subjects (HS) Fibromyalgia subjects (FS)	HS: 20 enrolled/19 final analysis FS: 8	HS: 10 FS: 60
Caumo et al., 2021	Double blind RCT	tDCS	Fibromyalgia	48	20
Charvet et al., 2017	Double blind RCT	tDCS	Multiple Sclerosis	Study 1: 15 active/20 sham Study 2: 15 active/12 sham	Study 1: 10 Study 2: 20
Garcia-Larrea et al., 2019	Double blind RCT	tDCS	Neuropathic Pain	12	35
Gulley et al., 2021	Double blind RCT	tDCS	Mild to moderate Alzheimer’s Related Dementia	100	130
Hyvärinen et al., 2016	Double blind RCT	tDCS	Tinnitus	35 (23 active/12 sham)	10
Im et al., 2019	Double blind RCT	tDCS	Early Alzheimer’s Related Dementia	18 (11 active/7 sham)	182
Mortensen et al., 2016	Double blind RCT	tDCS	Stroke-Patients with Upper Limb Motor Impairment following Intracerebral Hemorrhage	15 (8 active/7 sham)	5
Prathum et al., 2021	Double blind RCT	tDCS	Chronic Stroke	24 (active/sham distribution not specified)	12
Shaw et al., 2017	Double blind RCT	tDCS	Multiple Sclerosis (MS) and Parkinson’s Disease (PD)	Study 1: 26 (MS) Study 2: 20 (MS) and 6 (PD)	Study 1: 10 (MS) Study 2: 20 (MS) and 10 (PD)
Hagenacker et al., 2014	Double blind Cross-over RCT	tDCS	Trigeminal Neuralgia	17 enrolled, 10 final analysis	14
Martens et al., 2018	Double blind Cross-over RCT	tDCS	Minimally Conscious State	37 enrolled/27 Final analysis	2 × 20 (5/week)
O’Neill et al., 2018	Double blind Cross-over RCT	tDCS	Neuropathic Pain	21	5 active and 5 sham, with 4 week washout period in between.
André et al., 2016	Single blind RCT	tDCS	Mild Vascular Dementia	21 (13 active/8 sham)	4
Cha et al., 2016	Single blind RCT	tDCS	Mal Debarquement Syndrome	23 (12 active, 10 sham, 1 open label)	20 (5/week)

Firstly, the tDCS administration process was complex, while participants were only offered limited formal training. Secondly, placing the tDCS electrodes on the target location required the participant to perform complex measurements, increasing the risk of misplacement that might compromise the safety and efficacy of the intervention.

To overcome these limitations, we built on the recommendations of the International Federation of Clinical Neurophysiology for training in non-invasive brain stimulation (Fried et al., 2021) and the guidelines proposed by Charvet et al. (2015, 2020) for training home-tDCS users, to design an innovative home-based tDCS training program to empower a study companion (e.g., family member, spouse, or caregiver) to safely and effectively administer tDCS in the home environment. This program was completed entirely via training sessions conducted via video-conference, together with self-directed learning via video and paper-based material, and thus did not require participants or study companions to come into the laboratory. This was particularly advantageous given the COVID-19 pandemic, but is also beneficial outside of COVID. Moreover, we adopted a newly developed user-friendly home-based tDCS system that simplifies the administration process by reducing the possibility of electrode misplacement and incorporating a system of real-time monitoring with embedded notifications to optimize the safety and efficacy of tDCS intervention in the home setting. The purpose of the present study was to investigate the feasibility of this remotely supervised, study companion-administered tDCS intervention within the homes of older adults with diagnosed MDD.

## Materials and Methods

### Patient Participants

Demographic and clinical characteristics are presented in Table 2. All patient participants met criteria for a diagnosis of MDD according to the Diagnostic and Statistical Manual of Mental Disorders (DSM-IV-TR; American Psychiatric Association, 2000), as determined via a telehealth interview with the study psychiatrist. In order to be enrolled individuals had to: (1) have a score of at least 20 on the Montgomery–Åsberg Depression Rating Scale (MADRS; Montgomery and Åsberg, 1979) (in line with previous literature, Alonzo et al., 2019), (2) identify a primary psychiatrist who agreed to their participation in the study and was willing to continue to follow the patient and work collaboratively with the study team, (3) be assessed by their primary psychiatrist to be stable enough to remain at home and participate in the present study without undue risk to their safety, (4) be living with an adult willing and capable to provide oversight and learn to deliver the home-based tDCS, and (5) have the capability and willingness to commit to connect with the study team for daily supervision of the intervention sessions and close safety monitoring.

**TABLE 2:** Demographic and clinical characteristics of patient participants.

Study ID	MDD001	MDD002	MDD004	MDD005	MDD006
Age	72	70	56	46	72
Gender	Male	Male	Male	Male	Male
Race	White/Caucasian	White/Caucasian	White/Caucasian	White/Caucasian	White/Caucasian
Education	Bachelor’s degree	Master’s degree	Associate’s degree	High School	Master’s degree
Primary language	English	English	English	English	English
Handedness	Ambidextrous	Left	Right	Right	Right
Marital status	Married	Married	Legally Separated	Married	Married
Number of children	2	0	2	4	2
Job	Retired	Textile Industry	X-ray technician	Arborist	Insurance Industry
Currently employed?	Yes	No	No	Yes	Yes
Support network	Family	Family and Friends	Family and Friends	Family and Friends	Family and Friends
Trauma/abuse/neglect history	Yes	No	No	Yes	No
Self-injurious behavior	No	No	No	Yes	No
Suicidal ideation	No	No	Yes	Yes	No
Family psychiatric history	Yes	No	–	No	No
Diagnosis—depression	Yes	Yes	Yes	Yes	Yes
Diagnosis—bipolar disorder	No	No	No	No	No
Diagnosis—schizophrenia	No	No	No	No	No
Diagnosis—anxiety	No	No	Yes	Yes	Yes
Mood-related Medication	Methylphenidate, 10 mg daily	Rasagiline, 1 mg daily Carbidopa/levodopa, 300 mg, 3× per day	Quetiapine, 400 mg Bupropion, 150 mg Tranylcypromine, 60 mg daily	Quetiapine, 300 mg Paroxetine, 60 mg Buspirone, 20 mg daily	Sertraline, 100 mg Trazodone, 50 mg daily Gabapentin, 300 mg as needed

*All screened individuals were given a study ID. MDD003 is not reported here because this individual was screened but not enrolled into the study.*

Exclusion criteria were any DSM-IV-TR psychotic disorder; drug or alcohol abuse or dependence in the preceding 3 months; concurrent benzodiazepine medication; high suicide risk; history of clinically defined neurological disorder or insult; skull defects; skin lesions on the scalp at the proposed electrode sites; and pregnancy. Patient participants on antidepressant medications were permitted to enter the trial provided the medication dose had been unchanged for one month prior to trial entry. Consistent with prior tDCS studies, patient participants with a diagnosis of bipolar disorder were required to be on a mood stabilizer such as prophylaxis against treatment-emergent mania for at least 1 month prior to trial entry. Additional exclusion criteria in consideration of safety of tDCS included any cranial metal implants (excluding ≤1 mm thick epicranial titanium skull plates and dental fillings) or medical devices (i.e., cardiac pacemaker, deep brain stimulator, medication infusion pump, cochlear implant, vagus nerve stimulator).

### Study Companions

All study companions were required to be at least 21 years of age, able to read, write, and communicate in English, and have self-reported computer proficiency and willingness to learn how to use tDCS. Demographic characteristics and computer proficiency are presented in Table 3.

**TABLE 3:** Demographic characteristics and computer proficiency of study companions.

Study ID	SC001	SC002	SC004	SC005	SC006
Age	70	68	56	34	72
Gender	Female	Female	Male	Female	Female
Race	White/Caucasian	White/Caucasian	White/Caucasian	White/Caucasian	White/Caucasian
Education	Master’s degree	Three or more years of graduate school	Bachelor’s degree	Bachelor’s degree	Bachelor’s degree
Computer comfortability	Extremely comfortable	Extremely comfortable	Somewhat comfortable	Extremely comfortable	Somewhat comfortable

### Study Design

**FIGURE 1:**
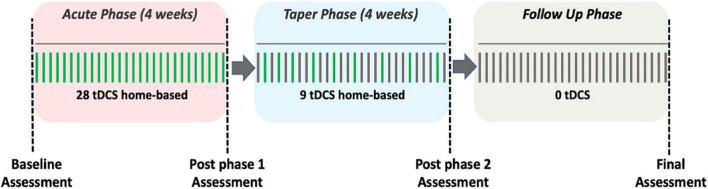
Study design. The green vertical lines represent a day with a scheduled tDCS session, whereas the gray vertical lines represent a day without a tDCS session.

The treatment course consisted of a total of 37, 30-min home-based tDCS sessions over 8 weeks, with a final follow-up assessment 1 month after the final tDCS session (Figure 1). The total study duration was 12 weeks. The protocol consisted of an acute phase, a taper phase, and a follow up phase each lasting 4 weeks. During the acute phase, the study companions conducted daily tDCS for a total of 28 sessions (i.e., 7 days a week). During the taper phase study companions completed a total of nine stimulation sessions. Specifically, the first of the three stimulation sessions were conducted every second day, the next three stimulation sessions were completed every third day, and the last three stimulation sessions were completed every fourth day. During the follow up phase no stimulation was administered. Assessments were conducted at baseline and at the end of each of the three phases. The purpose of the follow up phase was to capture the extent to which any effects of tDCS on mood may have been sustained after four weeks of no stimulation. Further, a research staff member called the study companion once a week during the acute and the taper phase to check on the patient participant’s condition and to record any subjective effects of stimulation on mood using a global impression scale. Prior to the start of the intervention protocol study companions underwent a training program to become competent in home-based tDCS administration.

### Study Procedures

The study was ethically reviewed and approved by Advarra review, and registered as a clinical trial at clinicaltrials.gov (NCT04799405). All subject interaction was conducted remotely via telehealth/videoconferencing media. Before enrolling into the study, patient participants and study companions attended a 45-min phone screening to ensure they fulfilled eligibility criteria. If eligible, subjects attended a second visit with the study psychiatrist during which they signed the informed consent form using Right Signature (©1999-2021 Citrix Systems, Inc.), a secure, web-based electronic document signature platform. Subsequently, the stimulation equipment was shipped to the patient participant’s home alongside the training educational materials. A research staff member scheduled the home-based tDCS sessions with the patient participants. Training in the administration of tDCS was then carried out remotely (please refer to the training program section below). The baseline assessment, the post phase I assessment, the post phase II assessment, and the final assessment were conducted over the phone with the study psychiatrist.

### tDCS Starstim Home Kit

The specific tDCS system used in this trial was the Starstim^®^-Home Kit (Neuroelectrics Corp.). The Starstim system includes a headcap that resembles a swimming cap with holes located where small electrodes can be attached and secured in place in the correct position on the scalp. These electrode holes are color- and number-coded so that electrode leads with corresponding colors coming from the tDCS device are appropriately attached to the corresponding electrodes, eliminating the potential for accidental mismatching of the electrodes and the leads. The Starstim^®^-Home Kit further incorporates a smart tablet wirelessly connected to the internet. This tablet allowed the study companions and patient participant to initiate the tDCS sessions, receive specific step-by-step instructions needed to complete the tDCS administration process, and record any side effects via custom-developed questionnaires completed with the tablet. The tablet automatically runs an impedance check before and during the delivery of the tDCS current, and blocks the stimulation if the electrode impedance reaches above 20 kΩ. Moreover, the tablet has a manual abort function for the participant to stop the stimulation if they are experiencing any discomfort or pain. The research staff are notified if this occurs and reach out to the participant to resolve the situation. The tablet further interfaces with another component of the Starstim^®^-Home Kit called the Neuroelectrics Portal which can be used by the research staff to schedule a specific time slot when the execution of the tDCS sessions is allowed. If the stimulation is attempted outside of this time slot, the tablet will inform the participant that the stimulation is currently unavailable and indicates when the next time slot is scheduled. The tablet further allows the study staff to remotely monitor patient participant progression through each session, side effects, and treatment compliance. This portal also ensures that all the stimulation parameters, including stimulation intensity, stimulation duration, and number of sessions, are pre-configured into the system and cannot be adjusted by study companions or patient participants.

### Optimized Current Flow Modeling to Target the Left Dorsal Lateral Prefrontal Cortex

**FIGURE 2:**
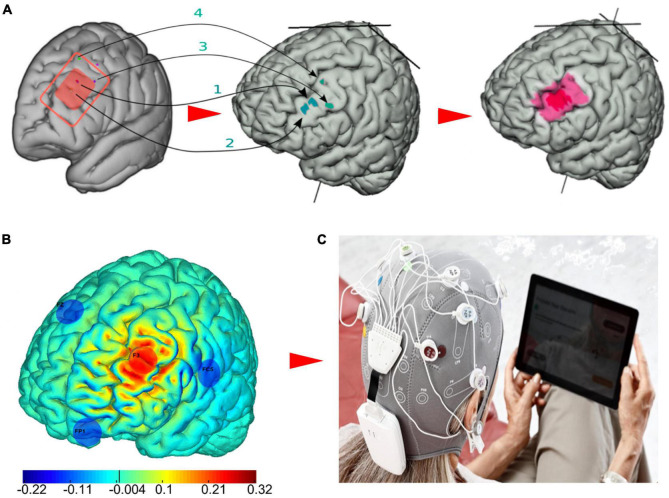
The multichannel tDCS intervention for major depressive disorder (MDD). **(A)** The red rectangle represents the left DLPFC, which is inclusive of evidence-based TMS targets for depression as reported by Fox et al. (2012) and the Beam F3 method (Trapp et al., 2020). First, the MNI coordinates [x,y,z] of the TMS hotspots (1: [−40.6, 41.7, 34.3; −41.5, 41.1, 33.4], 2: [39.3, 46.2 27.5; −41.3, 48.9, 27.7], 3:[−50, 30,36], 4: [−33.6, 30.8, 51.11]) were remapped on the cortex of the default brain model. Then, in order to obtain the final target map considered for this study, we drew an inner hotspot area encompassing all the mapped points and surrounded it by a buffer area. **(B)** The optimized four-electrode montage developed in this study to target the left DLPFC (anode shown in red, cathodes in blue) and normal component of the electric field to the cortex induced by the montage (V/m). **(C)** The Starstim^®^-Home Kit (Neuroelectrics Corp.) was used to administer stimulation.

The left dorsal lateral prefrontal cortex (L-DLPFC) has been consistently related to depression symptomatology (Mayberg, 2001; Pizzagalli, 2011). Specifically, the L-DLPFC is hypoactive in depression, and an increase in activity is associated with antidepressant response. Therefore, each session involved a multichannel tDCS montage with maximal anodal current targeting the left DPFC administered via 4 NG Pistim electrodes (circular electrodes with a contact of area of 3.14 cm^2^) using the Starstim^®^-Home system. Created using the Stimweaver^®^ algorithm (Ruffini et al., 2014), the montage was specifically designed to optimize anodal stimulation over the left DLPFC and at the same time minimize off target stimulation effects based on a standard brain model. The electrode positions were F3 (anode) as well as FZ, FC5, and FP1 (cathodes), according to the 10-20 EEG system (Figure 2). During each session a maximum current per electrode was ca. 1.75 mA, well below recommended safety limits (Antal et al., 2017). The average E-field normal component on the target was En = 0.11 V/m, with En = 0.19 V/m in the hotspot and En = 0.09 V/m on the surrounding region. In the rest of non-stimulated cortex, it remained low, En = −0.0001 V/m. For all patient participants, current intensity was ramped up over 30 s, then sustained at the stimulation intensity for 30 min, then ramped down over 30 s.

### HSL Home-Based Remotely Supervised tDCS Training Program

**FIGURE 3:**
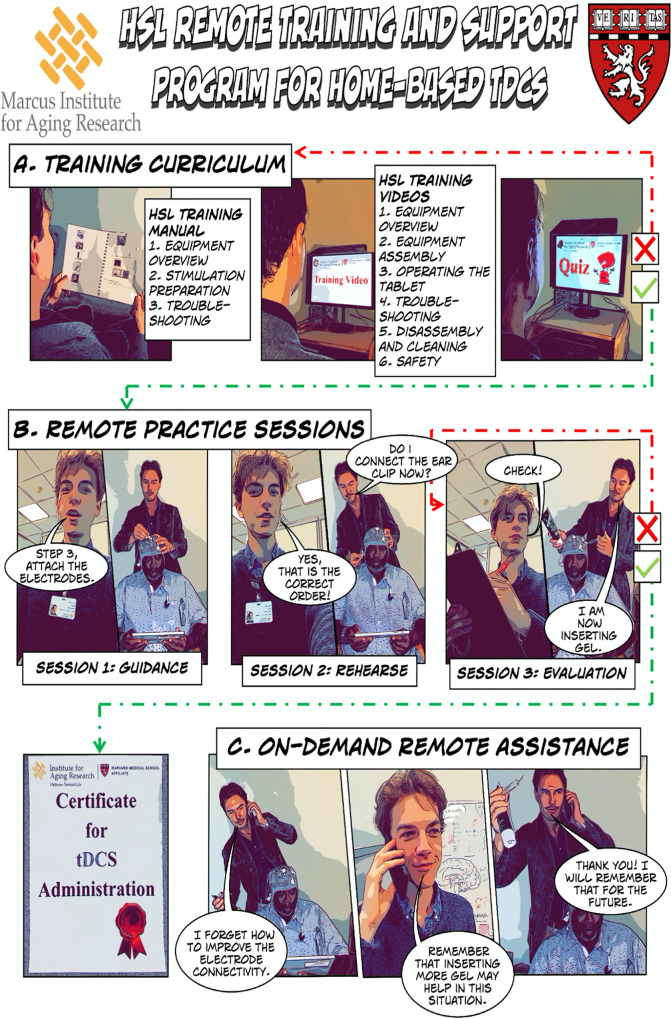
HSL Remote Training and Supervision Program for Home-Based tDCS. **(A)** Study companions are provided with a training curriculum for self-study. **(B)** The study companion attends practice sessions with an evaluation at the end. **(C)** Once certified in independent tDCS administration, study companions can access remote assistance during the home-based tDCS sessions. The images in this figure were created using Apple’s iOS 12 camera filter: Comic Book, and the other elements of the figure were created using Comic Life 3 by plasq LLC.

The Hebrew SeniorLife (HSL) remote training and supervision program for home-based tDCS is an innovative program designed to provide the study companions with a high level of knowledge and skill in the process of administering tDCS to a patient participant. The program is comprised of three main pillars: an in-depth training curriculum about tDCS and instructions on how to administer the stimulation, a set of remote practice sessions hosted by trained research staff for study companions to promptly apply the knowledge and skills derived from the training curriculum, and an on-demand remote assistance infrastructure to provide additional guidance to study companions as needed (see Figure 3).

#### Training Curriculum

The training curriculum consists of a written manual and a six-part video series with a set of embedded quizzes to test the success of knowledge acquisition (Figure 3A). The manual contains a summary of the stimulation equipment, step by step instructions on the preparation of the stimulation equipment, and concrete examples of troubleshooting during the stimulation session. The six-part video series elaborates on the components included in the home-based stimulation kit, the operations involved in the assembly of the stimulation equipment, the handling of the Starstim^®^-Home smart tablet, how to troubleshoot problems with scalp-electrode connectivity, useful tips for how to clean and care for the stimulation equipment, and on how study personnel safeguard patient participant wellbeing throughout the intervention. Study companions are then presented with a set of rigorous interactive assessments, in the form of short web-based quizzes that are embedded at the end of each video in the series to assess the study companion’s comprehension and retention of the knowledge provided. The study companion must achieve a score of at least 80% on each of the quizzes to be allowed to proceed to the next phase of the training program.

#### Remote Practice Sessions

Having built a comprehensive foundational understanding of how to carry out home-based tDCS, study companions are invited to apply their newly gained knowledge by attending a set of remote practice sessions. During these sessions, a research staff member connected with the study companion and patient participant over Zoom (v5.8.4. ©2012-2021 Zoom Video Communications, Inc.) to guide them through the home-based tDCS administration process. The research staff member facilitates the translation of the knowledge obtained into practical skill and provides additional tips on how to appropriately handle the stimulation equipment, achieve good connectivity between the scalp and the electrodes, and ensure patient participant comfort. A minimum of three remote practice sessions are provided to the study companion. During the first session, the research staff member instructs the study companion step by step on how to administer the tDCS. During the second session, the study companion is encouraged to carry out as much of the session as possible without instructions from the research staff. However, the study companion was free to ask for assistance where needed, and research staff corrects them should they commit any errors. During the third session, the study companion conducts the tDCS without any instruction from the research staff and the administrator’s performance is assessed using an evaluation check list. The check list is comprised of all the specific steps that need to be carried out to successfully conduct the home-based administration of tDCS. Only once the study companion demonstrates the ability to successfully conduct each of the operations on this list, do they receive the certification to conduct the tDCS administration process independently (see left bottom of Figure 3). If the study companion does not pass the evaluation check, or the study companion expresses a lack of comfort in carrying out the tDCS session on their own, an additional practice session is scheduled for the next day. We adopted the checklist previously published by Charvet et al. (2015).

#### On Demand Remote Assistance

Once the study companion successfully completes the first two phases of the training program, they are credentialed to carry out the home-based tDCS sessions independently. The research staff automatically receive an email notification as the study companion initiates and carries out a tDCS session. Study companions are encouraged to contact research staff for assistance should any issues, technical or otherwise, arise that the administrator cannot solve on their own. One of the research staff members responds within 60 min. First, assistance is offered over the phone or via email. If this is unsuccessful, the research staff member invites the study companion to connect over video call to visually elaborate on the problem. Once the issue is solved, the research staff member explicitly describes to the study companion how this problem can be avoided in the future. We developed specific standardized scripts to explain in lay language how to prevent or fix the issue.

#### Study Outcomes

##### Feasibility

The compliance to the treatment schedule was used to evaluate the feasibility of the home-based tDCS protocol. Two metrics were recorded to determine the effectiveness of the training program. Firstly, since the successful decreasing of electrode impedance is a crucial aspect of being skillful at tDCS administration, the number of sessions during which stimulation was aborted due to high electrode impedance was recorded. Secondly, because a successful training process should decrease study companions’ need for study staff assistance, the frequency with which study companions required remote technical assistance was recorded.

##### Safety

Before and after each home-based tDCS session, patient participants were asked to report any side effects on questionnaires that were implemented using the Starstim^®^-Home system smart tablet. Patient participants were asked to report whether any of a set of physical sensations, scalp abnormalities, and suicidal thoughts were present. The specific side effects that patient participants were asked about are reported in the results section. Patient participants were asked to report on the intensity of the side effects, by stating whether their experience for each of the side effects was: absent, mild, moderate, or severe. The responses were received by study staff via a study email inbox in real-time. If a patient participant reported experiencing a side effect of a moderate or severe intensity, this was automatically classified as an adverse event, a special SMS-based alerting system was activated to notify the research staff. Specific SOPs were activated by study staff to safeguard patient participant wellbeing. Furthermore, if patient participants indicated a moderate or severe experience of a side effect prior to the tDCS, access to the stimulation was blocked until study staff could ensure that the patient participant was fit for stimulation.

##### Clinical Outcomes

Clinical outcomes included in the trial focused on mood, quality of life, and cognitive function. They were assessed at baseline, after the acute phase, after the taper phase, and at the 1-month follow-up (see Table 4), via telehealth interview with the study psychiatrist. Data were acquired and stored using a REDCap database, a secure virtual platform for storing data and generating reports.

**TABLE 4:** Clinical outcome scores.

	Baseline	Post phase 1	Post phase 2	Follow up	% Change from baseline
**MADRS**					
*MDD002*	28	11	0	6	–78.57
*MDD004*	44	29	21	25	–43.18
*MDD005*	38	11	10	17	–55.26
**QIDS-SR16**					
*MDD002*	15	3	0	0	–100
*MDD004*	26	14	16	10	–61.54
*MDD005*	18	5	11	6	–66.67
**HDRS**					
*MDD002*	17	10	3	1	–94.12
*MDD004*	23	16	23	14	–39.13
*MDD005*	21	6	18	7	–66.67
**BDI-II**					
*MDD002*	21	8	1	2	–90.48
*MDD004*	36	25	29	18	–50
*MDD005*	24	10	14	11	–54.17
**Q-LES-Q-SF**					
*MDD002*	34	50	53	48	41.18
*MDD004*	31	36	35	44	41.94
*MDD005*	36	44	37	42	16.67
**MoCA**					
*MDD002*	22	27	29	28	27.27
*MDD004*	29	30	28	28	–3.45
*MDD005*	29	28	30	30	3.45

*MADRS, Montgomery; QIDS-SR16, Quick Inventory of Depressive Symptomology–Self-Report; HDRS, Hamilton Depression Rating Scale 17-item; BDI-II, Beck Depression Inventory; Q-LES-Q-SF The Quality of Life Enjoyment and Satisfaction Questionnaire—Short Form, MoCA Montreal Cognitive Assessment.*

#### Primary Clinical Outcome

##### Montgomery–Åsberg Depression Rating Scale

Is a clinician-rated scale which consists of 10 items; each item is rated on a 0–6 scale, resulting in a maximum total score of 60 points, with higher scores indicative of greater depressive symptomology (Montgomery and Åsberg, 1979). The MADRS total score ranging from 0 to 6 indicates no depression, a score ranging from 7 to 19 indicates mild depression, 20 to 34 indicates moderate depression, a score of 35 and greater indicates severe depression, and a total score of 60 or greater indicates very severe depression. The primary outcome was the change in the MADRS score from baseline to the 1-month follow-up. Clinical response was defined as ≥50% improvement in MADRS score from baseline to the 1-month follow up. Remission was defined as MADRS score ≤10.

#### Secondary Clinical Outcomes

##### Mood

The secondary outcome measures included the Beck Depression Inventory (BDI-II; Beck et al., 1996) and Hamilton Depression Rating Scale (HDRS; Hamilton, 1986) and the patient participant-rated Quick Inventory of Depressive Symptomatology (QIDS-SR; Rush et al., 2003).

##### Quality of Life

The Quality of Life Enjoyment and Satisfaction Questionnaire Short Form (Q-LES-Q-SF; Endicott et al., 1993) was also administered at the same time points as the MADRS and other secondary mood outcomes.

##### Cognitive

Parallel versions the non-visual phone MoCA (Wittich et al., 2010) and digit span (Wechsler, 1997) tests, were administered over the phone to provide an additional safety outcome measure.

## Results

### Feasibility

Five patient participants were enrolled into the study (Table 2). Two withdrew from the study during the first week of tDCS sessions due to medical conditions unrelated to study treatment (one patient participant became preoccupied with other health issues and one tDCS study companion developed health issues that interfered with their ability to participate). Out of a total of 111 tDCS sessions scheduled for the remaining three patient participants, just one session was missed, and all study assessment points were successfully completed. The training program was well-received by the three study companions and all three were able to independently and safely administer the tDCS after the three planned practice sessions. Less than 10% of home-based sessions were aborted due to impedance issues and the study companions were able to resolve the issue and correctly complete the aborted sessions. Study companions required remote assistance from a research staff member on just four occasions (i.e., 3.6% of sessions), indicating that the training empowered the study companions to independently solve technical challenges related to tDCS administration.

### Safety

All reported side effects were mild and transient (Table 5). The most frequently reported side effects were sensations under the electrodes such as tingling and itching, post-stimulation sleepiness, scalp redness, and neck pain. Neck pain was reported by only one patient participant and was due to an unrelated chronic condition that was not exacerbated by the intervention. No session was aborted due to discomfort or pain. There were no other adverse events reported over the course of the intervention.

**TABLE 5:** Total incidence of side effects and their severity in 110 home-based tDCS sessions.

Side effect	Mild	Moderate	Severe	Percentage of sessions
Headache	0	0	0	0
Neck pain	34*	0	0	29.82
Scalp pain	0	0	0	0
Sensations under electrodes	21	0	0	18.42
Sleepiness	4	0	0	3.51
Scalp burn	0	0	0	0
Scalp redness	1	0	0	0.88
Increase in suicidality	0	0	0	0

*All responses derived from MDD04.

#### Primary Clinical Outcome

##### Montgomery–Åsberg Depression Rating Scale

**FIGURE 4:**
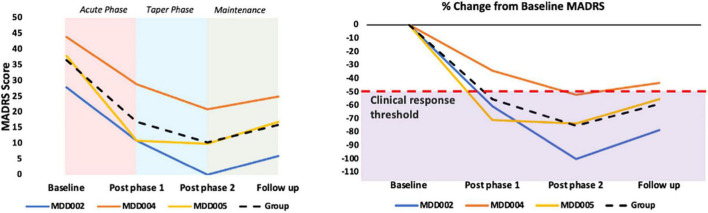
Primary clinical outcome: MADRS results. The purple shaded area in the graph B represents a ≥50% decrease in the MADRS score, which is used as a clinical response threshold.

All three completers showed beneficial effects as captured by the MADRS, with an average decrease of 59% in the MADRS score between baseline and the 1-month follow up. Individual results are provided in Figure 4 and Table 4.

#### Secondary Clinical Outcome

##### Depressive Symptomatology

The scores on the secondary mood outcomes, QIDS-SR16, HDRS, and BDI, followed the same trend as the MADRS scores. Specifically, an average decrease of 76% was observed at the follow up assessment as compared to baseline for the QIDS-SR16, an average decrease of 67% for the HDRS, and an average decrease of 65% for the BDI.

##### Quality of Life

The average score on the Q-LES-Q-SF increased by 33% from baseline to the follow up assessment, indicating a substantial improvement in quality of life.

##### Global Cognitive Functioning

No changes in MoCA score were observed during the intervention in two of the patient participants, but MDD02 showed a 5-point improvement during the intervention, largely because of an improvement in verbal delayed recall from 0 at baseline to 5 at the end of phase 2 assessment. The only changes in digit span performance between baseline and the final assessment were an increased digit span forward score by 1 point for MDD002, and an increased digit span backward score by 1 point for MDD004.

## Discussion

This paper describes a novel protocol for a remotely-supervised study companion-administered home-based tDCS intervention and indicates its feasibility and safety. A crucial aspect of the protocol is a novel training program that includes training materials for self-study, remote practice sessions with research staff, and on-demand remote assistance that successfully trained study companions to administer tDCS safely and effectively. None of the study companions had prior tDCS experience, and all were older adults (age range 46–70). The training program is particularly valuable for the field of non-invasive neuromodulation, as this program could be adapted to train study companions to administer home-based tDCS as interventions for possible applications.

The training program was successful in providing study companions with a high level of knowledge and skill in the home-based tDCS administration process. The program was carefully designed following the recommendations of the training guidelines endorsed by the International Federation of Clinical Neurophysiology (Fried et al., 2021) as well as the recommendations put forth by Charvet et al. (2015, 2020) regarding standards for training of home-users of tDCS, and based on the knowledge acquired from the administration of hundreds of tDCS sessions across diverse clinical populations and multiple studies. We think that the combination of first a self-study phase with tailored training curriculum materials followed by an opportunity to apply this knowledge in a set of practice sessions expedited the learning process. By embedding quizzes at the end of each video section, we ensured that study companions had achieved a high level of understanding of the home-based tDCS administration process, the equipment, and the safety protocols that could be built upon in the remote practice sessions. The research staff was able to assert that study companions displayed a substantial level of skill even during the first practice session. Specifically, study companions were promptly able to follow the instructions of the research staff members, even when they consisted of complicated operations involving equipment with a complex nomenclature. The study companions themselves were positive about the training program and reported that they were confident in their own ability to independently administer the tDCS. This was exemplified by the study companions requiring remote assistance on only 4 occasions throughout the study, a mere 3.6% of all sessions. This is promising for future research as it denotes that the training program can free up research staff from having to technically assist study companions in the home-based tDCS administration process, substantially increasing the intervention’s scalability.

There has been a growing interest to employ home-based tDCS as an intervention for a wide array of clinical conditions, research so far has been done on conditions ranging from neuropathic pain (Garcia-Larrea et al., 2019) to Multiple Sclerosis (Charvet et al., 2017). The described remote training program could be adapted to clinical contexts beyond depression. However, further studies are needed about the translatability of the training program across pathologies and tDCS methods and protocols. The ease of use and connectivity of the home stimulation solution (Starstim^®^-Home) we employed was another key element of the study.

A limitation of previous home-based tDCS studies was the challenge in monitoring the occurrence of expected and unexpected side effects. We overcame this limitation with the use of the Starstim^®^-Home system which allowed us to effectively monitor side effects throughout the trial. No serious adverse events occurred across the total of 110 home-based tDCS sessions. This is a testament also to the efficacy of the training program and in line with previous studies that applied tDCS as an intervention for major depression which found that tDCS was well-tolerated, that no significant adverse events or side effects were reported, and that active tDCS had a side effect profile comparable to sham (Brunoni et al., 2017; Alonzo et al., 2019; Moffa et al., 2020; Razza et al., 2020). In our feasibility pilot study, the side effects reported during the acute, taper, and follow-up phases were minimal, mild, and transient. Specifically, on an intensity rating with a range of absent, mild, moderate, to severe, patient participants never reported any side effects above mild. No negative effects on cognition were observed, with MoCA scores remaining stable across the trial duration (and even increasing by 5 points for one patient participant, MDD002).

Although two patient participants withdrew from the study in the first week, both dropouts were due to factors external to the intervention and thus do not reflect negatively on the safety profile of the protocol nor the intervention. We would not expect any significant difference in withdrawal rates in other types of tDCS intervention, such as clinic-administered tDCS or tDCS administered at home by visiting medical personnel.

One limitation of this study is that the majority of the patient participants and study companions in this trial hold degree-level qualifications and are Caucasian. Therefore, the authors encourage future feasibility studies with more diverse groups of patient participants and study companions.

Further, the clinical results presented here have to be taken with great caution as they come from only three patient participants enrolled in an open label study that was designed to evaluate the feasibility and safety of our protocol. However, it is worth noting that all three completers reached the criterion for clinical response (≥50% decrease in MADRS score relative to baseline) at one or more of the follow up assessments, and that there was an average decrease of 59% in the MADRS score at the 1-month follow up relative to baseline. These findings are in line with previous literature demonstrating clinically beneficial effects of home-based and lab-based tDCS for major depression (Alonzo et al., 2019; Moffa et al., 2020; Razza et al., 2020).

## Conclusion

This study has demonstrated the feasibility of an innovative home-based, remotely supervised, study companion-led, multi-channel tDCS intervention for older adults suffering from MDD, and introduces a novel and reliable training curriculum for remote instruction of study companions in the administration of tDCS. The training program presented has proven effective in empowering tDCS study companions to quickly develop comfort and competency of intervention procedures. While additional data from this feasibility study as well as future controlled trials will be needed to determine the effectiveness of the home-based intervention in MDD, it is encouraging that all three completers tolerated the intervention, reported no serious adverse events or side effects, and showed improvements within the study’s primary and several secondary endpoints.

## Data Availability Statement

The raw data supporting the conclusions of this article will be made available by the authors, without undue reservation.

## Ethics Statement

The studies involving human participants were reviewed and approved by Advarra Review. The patients/participants provided their written informed consent to participate in this study. Written informed consent was obtained from the individual(s) for the publication of any potentially identifiable images or data included in this article.

## Author Contributions

DC: conceptualization, methodology, writing of original manuscript draft, supervision, analysis, and interpretation of data. TB: conceptualization, methodology, writing of original manuscript draft, acquisition of data, analysis, and interpretation of data. CJ: acquisition of data and reviewing and editing of manuscript. WY, AL, NL, OM, EM, and BM: reviewing and editing of manuscript. MB: creating stimulation model and reviewing and editing of manuscript. PS: technical support and reviewing and editing of manuscript. GR: creating stimulation model and reviewing and editing of manuscript. AP-L: conceptualization, methodology, reviewing and editing of manuscript, supervision, analysis, and interpretation of data. All authors contributed to the article and approved the submitted version.

## Conflict of Interest

MB and PS were employed by company Neuroelectrics. GR is a shareholder and works for Neuroelectrics. AP-L is a co-founder of Linus Health and TI Solutions AG; serves on the scientific advisory boards for Starlab Neuroscience, Magstim Inc., Radiant Hearts, and MedRhythms; and is listed as an inventor on several issued and pending patents on the real-time integration of non-invasive brain stimulation with electroencephalography and magnetic resonance imaging. The remaining authors declare that the research was conducted in the absence of any commercial or financial relationships that could be construed as a potential conflict of interest.

## Publisher’s Note

All claims expressed in this article are solely those of the authors and do not necessarily represent those of their affiliated organizations, or those of the publisher, the editors and the reviewers. Any product that may be evaluated in this article, or claim that may be made by its manufacturer, is not guaranteed or endorsed by the publisher.
